# Neurocognitive Disorder and Emotional Symptoms in HIV+ Brazilian Elderly: Influence of Gender, Income, Diet, and Sleep

**DOI:** 10.3389/fnhum.2021.721029

**Published:** 2021-09-14

**Authors:** Sabrina Martins Barroso, Kelly Cristina Ramira Sousa

**Affiliations:** ^1^Psychological Assessment and Health Investigations Center, Department of Psychology, Federal University of Triângulo Mineiro, Uberaba, Brazil; ^2^Psychological Assessment and Health Investigations Center, Federal University of Triângulo Mineiro, Uberaba, Brazil

**Keywords:** human immunodeficiency virus (HIV), elderly, antiretroviral treatment (ART), cognitive deficits, HIV-associated neurocognitive disorder (HAND), anxiety, depression

## Abstract

The purpose of this study was to identify factors associated with HIV-associated neurocognitive disorder (HAND) and symptoms of anxiety and depression in HIV+ Brazilian elderly on antiretroviral treatments. The study included 112 HIV+ elderly who completed a questionnaire, tests for cognitive screening, attention, problem solving, processing speed, visual perception, memory, and anxiety and depression scales. The results showed presence of HAND (89.3%), pathological anxiety (48.2%) and depression (58%) in the sample. Higher income was a protective factor for HAND (OR = 0.33). Waking up well-rested (OR = 0.63) and better diet quality (OR = 0.62) reduced the chance of pathological anxiety. Higher education (OR = 0.74) and waking up well-rested (OR = 0.61) reduced the chance of depression. Being female (OR = 7.73) increased the chance of depression. It can be concluded that it is important to evaluate cognitive and emotional aspects of HIV+ elders and to consider social and educational status, diet, and sleep in interventions, paying special attention to elderly women.

## Introduction

The evolution of antiretroviral treatments has contributed to increase the quality and life expectancy of HIV+ people since their emergence, changing their status from rapidly fatal to chronic. But this population still has lower life expectancy than the general population, making HIV+ people to be considered elderly from the age of 50 (Luther and Wilkin, 2007; Pio et al., 2017).

Between the emergence of the first case and the year 2020 Brazil registered 1,011,617 HIV+ people, of which 135,907 were elderly (Brasil, 2020). The elders are the population with the highest increase in HIV+ incidence in Brazil, with a 103% increase in new registrations observed between 2007 and 2017 (Brasil, 2018). Some aspects associated with this increase include the aging of the population, late diagnosis, misinformation of the elderly about forms of infection, and the increased life expectancy of those infected (Wallace et al., 2017).

As one ages there is a greater chance of the emergence of cognitive deficits (Ávila-Funes et al., 2016) and anxious and depressive emotional symptoms (Fialho et al., 2017). In HIV+ people this risk increases, since clinical impairments, such as the presence of the HIV in the Central Nervous System and opportunistic diseases, can trigger or worsen cognitive deficits (Caliari et al., 2017; Medeiros Júnior et al., 2019) and the side effects of the antiretroviral treatments itself has already been associated with a higher risk of neurological and cognitive damage (Vergara-Moragues et al., 2010; Giunta et al., 2011; Heaton et al., 2011; Azevedo et al., 2014). In Brazil, in addition to these risks, the living conditions of HIV+ elderly people need to be considered, because most people with HIV in the country are below the poverty line (Oliveira et al., 2017; Brasil, 2018, 2020).

In HIV+ people, the presence of deficits in two or more cognitive skills simultaneously is the condition for identifying HIV-associated neurocognitive disorder (HAND) (Anderson et al., 2016). The most frequent deficits in HAND occur in executive function, memory, information processing speed, attention/working memory, motor skills, language/verbal fluency, and sensoriperception (Cattie et al., 2012; Fialho et al., 2016).

Cognitive deficits worsen treatment adherence and increase the chance of early onset of opportunistic diseases and occurrence of risky sexual behavior (Gárcia-Torres et al., 2015; Fialho et al., 2016; Pio et al., 2017), which demonstrates the importance of identifying them, especially in the elderly population. The presence of depression and anxiety disorders also contribute to lower treatment adherence and poorer quality of life in HIV+ people (Nanni et al., 2015; Athar et al., 2017; Caliari et al., 2017; Pio et al., 2017; Reis et al., 2017; Medeiros Júnior et al., 2019).

The first studies on cognitive deficits and emotional symptoms in HIV+ Brazilian elderly began in the 2000s, but are still scarce (Kalil et al., 2009; Christo, 2010; Nascimento et al., 2015; Pinheiro, 2016; Oliveira et al., 2017). Considering this context, the purpose of this study was to identify factors associated with HAND and symptoms of anxiety and depression in HIV+ Brazilian elderly people on ART. The study’s hypotheses were that viral load, longer time since diagnosis, less education and worse eating and sleeping conditions would be associated with the presence of cognitive deficits in this population.

## Methods

### Sample

In this study, 112 HIV+ elderly people (50 years or older), who had been on ART for 6 months or more in a reference service for infectious diseases in a medium-sized city in the state of Minas Gerais, Brazil, were evaluated. The project followed the ethical precepts for research with human beings and was approved by the Research Ethics Committee (CAAE 01355018.40000.5154). A pilot study was initially conducted with five HIV+ elders to adapt the questionnaire and train the research team that conducted the collection. The evaluations were individual and occurred between August 2018 and March 2019.

The elderly had a mean age of 55.7 years (SD + 5.25), HIV+ diagnosis for 12.12 years (SD 6.99, minimum 1 and maximum 28 years), and CD4 load of 226.31 (SD = 346.70). Most of the participants were male (50.9%), self-reported as white (55.4%), single (40.2%), with family income of 1 to 2 minimum wages (73.2%; US$ 249.50 to 499.00), never studied (55.4%), and did not use other drugs except ART (53.6%) (Table 1).

**TABLE 1:** Characterization of the sample (*N* = 112).

**Variable**	***n***	**%**
**Sex**		
Female	55	49.1
Male	57	50.9
**Age group**		
50–59	67	59.9
60–69	37	33.0
70–79	08	7.1
**Color**		
White	62	55.4
Black	12	10.7
Yellow	05	4.5
Brown	31	27.7
Indigenous	02	1.8
**Marital status**		
Single	45	40.2
Male	20	17.9
Widowed	18	16.1
Divorced	23	20.5
Common-law marriage	06	5.4
**Education**		
Post-graduation	03	2.7
Higher education (complete and incomplete)	11	9.9
High school (complete and incomplete)	23	20.5
Elementary school (complete and incomplete)	13	11.6
Never studied	62	55.4
**CD4**		
<200	67	59.8
200+	45	40.2
**Family income**		
Over 5 minimum wages^1^	06	5.4
3 to 4 minimum wages	24	21.4
1 to 2 minimum wages	82	73.2
**Drugs**		
No	60	53.6
Yes	52	46.4
**Physical activity**		
No	63	56.3
Yes	49	43.8
**Meals**		
2 or less meals	23	20.5
3 meals	31	27.7
4 meals	37	33.0
5 or more meals	21	18.8
**Diet**		
Poor/very inadequate	08	7.1
Bad/inadequate	01	0.9
Regular	39	34.8
Good/adequate	54	48.2
Excellent	10	8.9
**Sleep**		
3 h or less	06	5.4
Between 4 and 5 h	22	19.6
Between 6 and 7 h	43	38.4
8 or more hours	41	36.6
**Rest**		
Never	18	16.1
A few times	25	22.3
Half of the time	15	13.4
Often	18	16.1
**Always**	36	32.1
Alcoholic beverage		
No	69	61.6
Yes	43	38.4
**Frequency of alcoholic beverage**		
Only once	04	9.3
At least once a month	11	25.6
At least once/week	25	58.1
More than once/week	02	4.7
At least once/day	01	2.3
**Illicit drugs**		
No	106	94.6
Yes	06	5.4
**Frequency of illicit drugs**		
Only once	05	83.3
At least once/week	01	16.7
**Cigarette**		
No	90	80.4
Yes	12	19.6

*^1^The value of the minimum wage was R$998.00 at the time of data collection, which was equivalent to US$249.50.*

### Instruments

A questionnaire with demographic and socioeconomic questions, time of diagnosis, use of medication, physical activity, frequency and perception of diet and sleep quality, use of alcoholic beverages and illicit drugs, was used. The following were assessed: functions for basic cognitive screening (Mini Mental State Examination; Bertolucci et al., 1994), problem solving and processing speed (Non-verbal Test of General Intelligence Beta – III; Rabelo et al., 2017), attention (Five Digits Test; De Paula et al., 2017) and visual perception, planning and visual memory (Rey Complex Figures; Oliveira et al., 2004). Two or more lower scores/deficits in different functions indicated presence of HAND.

Anxiety was evaluated using the Geriatric Anxiety Inventory (Martiny et al., 2011), with a score of 8–9 indicating high anxiety and 10 > pathological anxiety (Massena et al., 2015). Screening for depression was done by the Geriatric Depression Scale, 5-item version (Almeida, 2010), in which a score of 2 > indicated positive screening for depression. All tests were validated for Brazil.

All instruments were validated for Brazilian elderly people. To define the deficits, the values standardized by the authors of the instruments to identify deficits in elderlies were adopted.

### Data Analysis

Descriptive analyses were adopted and in the face of non-normality of cognitive variables (Shapiro–Wilk < 0.001), non-parametric two-way analyses (chi-square or Kruskal–Wallis) were used and separate robust logistic regressions were conducted for HAND, anxiety, and depression (initial models with variables with *p*-value up to 0.20 and final of 0.05) adopting Enter Method. For HAND, eight multivariate models were tested. The first model included the variables: sex, marital status, education level, family income, CD4, hours of sleep, waking up rested, drinking alcohol, and depression. For anxiety we tested 12 models. The initial model included: sex, family income, skin color, education level, HAND, CD4, diet quality, waking up rested and the test results for Five Digits Test, and Rey Complex Figures – copy. And for depression seven models were tested. The first model included: sex, wake up rested, family income, meals, diet quality, alcohol beverage, drugs, and Rey Complex Figures – copy. Model fit was based on Nagelkerke’s R^2^, Wald test, and Hosmer–Lemeshow test. The analyses were performed in IBM SPSS version 22.

## Results

HIV+ elderly had between 3 and 4 meals a day (60.7%) and considered their own diet as good/adequate or excellent (57.1%) (Table 1). Most of the elderly did not ingest alcoholic beverages (61.6%), illicit drugs (94.6%), did not smoke (80.4%), and did not engage in physical activity (56.3%). Many slept 6–7 h a night (38.4%) and considered that they always woke up well-rested (32.1%).

Most of the elderly showed a normal result in cognitive screening (56.3%), but there was inferior performance for processing speed for 82.1% and visual memory (80.4%) (Table 2). In attentional processes there was inferior performance ranging from 36.6% for Counting, to 50% for Alternating. Almost half of the sample underperformed for problem solving ability (44.6%) and visual perception (49.1%). The mood scales showed almost half of the sample (48.2%) with pathological anxiety and 58% with depression.

**TABLE 2:** Results of cognitive tests and mood check scales (*N* = 112).

**Test**	***n***	**%**
**Mini-Mental State Examination (Mean = 22.81; SD = 6.04)**		
Normal	63	56.3
Deficit	49	43.8
**Beta III – Codes (Mean = 14.58; SD = 15.16)**		
Upper	02	1.8
Upper middle	01	0.9
Middle	06	5.4
Lower middle	11	9.8
Lower	92	82.1
**Beta III – Matrix Reasoning (Mean = 16.70; SD = 17.48)**		
Upper	05	4.5
Middle	14	12.5
Lower middle	43	38.4
Lower	50	44.6
**Five Digits – Reading (Mean = 21.08; SD = 19.32)**		
Upper	05	4.5
Middle	14	12.5
Lower middle	43	38.4
Lower	50	44.6
**Five Digits – Counting (Mean = 27.28; SD = 24.54)**		
Upper	13	11.6
Upper middle	01	0.9
Middle	16	14.3
Lower middle	41	36.6
Lower	41	36.6
**Five Digits – Choice (Mean = 22.95; SD = 24.44)**		
Upper	10	8.9
Middle	13	11.6
Lower middle	35	31.3
Lower	54	48.2
**Five Digits – Alternation (Mean = 22.12; SD = 23.51)**		
Upper	09	8.0
Middle	15	13.4
Lower middle	32	28.6
Lower	56	50.0
**Five Digits – Inhibition (Mean = 33.88; SD = 33.40)**		
Upper	26	23.2
Middle	16	14.3
Lower middle	23	20.5
Lower	47	42.0
**Five Digits – Flexibility (Mean = 29.51; SD = 29.95)**		
Upper	19	17.0
Middle	09	8.0
Lower middle	42	37.5
Lower	42	37.5
**Rey Complex Figures – Copy (Mean = 39.15; SD = 36.95)**		
Upper	28	25.0
Upper middle	18	16.1
Middle	06	5.4
Lower middle	05	4.5
Lower	55	49.1
**Rey Complex Figures – Evocation (Mean = 15.56; SD = 18.83)**		
Upper	02	1.8
Upper middle	05	4.5
Middle	06	5.4
Lower middle	09	8.0
Lower	90	80.4
**HIV-associated neurocognitive disorders**		
Negative screening for HAND	12	10.7
Positive screening for HAND	100	89.3
**Geriatric Anxiety Scale (Mean = 10.19; SD = 6.22)**		
Mild to moderate anxiety	46	41.1
High anxiety	12	10.7
Pathological anxiety	54	48.2
**Geriatric Depression Scale (Mean = 1.96; SD = 1.45)**		
Negative screening for depression	47	42.0
Positive screening for depression	65	58.0

On the emotional aspects (Table 3), women (χ^2^ = 25.10; *p* < 0.001) and people with lower income showed more results for depression (KW = 8.83; p = 0.012). People with worse perceptions about their diet showed higher anxiety (KW = 10.37; *p* = 0.035) and more results for depression (KW = 12.76; *p* = 0.013). People who slept fewer hours per day (KW = 10.18; *p* = 0.017) and felt well-rested fewer days per week had higher anxiety (KW = 17.44; *p* = 0.002) and showed more results for depression (KW = 16.82; *p* = 0.002).

**TABLE 3:** *P*-values of one-way analyses of sample characteristics and emotional state with cognitive performance of HIV+ elders.

	**MMSE^1^**	**Beta III matrix reasoning**	**Beta III code**	**FDT reading^2^**	**FDT counting**	**FDT choice**	**FDT alternation**	**FDT inhibition**	**FDT flex^3^**	**FCR copy^4^**	**FCR evocation**	**HAND**	**Anxiety**	**Depression**
Sex	0.721	**0.033**	0.430	0.077	0.070	0.219	0.953	0.873	0.282	**0.003**	0.275	0.017	0.103	**<0.001**
Age group	0.857	0.118	0.852	0.242	0.423	0.494	0.207	0.072	0.807	0.995	0.835	0.533	0.595	0.428
Race/Color	0.516	0.473	0.407	**0.053**	**0.041**	0.318	0.143	0.205	0.156	0.706	0.562	0.521	0.942	0.179
Marital status	0.268	0.850	0.081	0.122	0.103	0.377	0.352	0.193	0.227	0.370	0.414	0.214	0.329	0.273
Education	**0.006**	**0.019**	0.481	0.154	**0.005**	0.002	**0.019**	**0.037**	0.182	0.018	0.175	0.091	0.588	0.192
Income	0.304	0.608	0.119	**0.055**	0.157	0.110	0.062	0.357	0.105	**0.001**	0.092	**0.023**	0.165	**0.012**
CD4	0.296	0.797	0.212	0.108	0.176	**0.035**	0.610	**0.012**	0.226	0.515	0.294	0.256	0.876	0.108
Time of diagnosis	0.321	0.584	0.916	0.996	0.635	0.933	0.636	0.110	0.145	0.719	0.515	0.734	0.842	0.519
Drug	0.633	0.566	0.419	0.546	0.500	0.254	0.345	0.753	0.650	0.954	0.446	0.726	0.467	0.484
Physical activity	0.549	0.434	0.206	**0.038**	0.267	0.489	0.213	0.067	0.461	0.354	0.283	0.281	0.895	0.866
Meals	0.277	0.186	0.981	0.853	0.167	0.174	0.106	0.266	0.316	0.980	0.209	0.724	0.123	0.507
Quality of diet	0.178	0.404	0.332	0.643	0.937	0.699	0.392	0.562	0.088	**0.030**	0.832	0.533	**0.035**	**0.013**
Sleeping hours	0.332	0.747	0.154	0.084	0.447	0.334	**0.034**	0.118	0.096	0.801	0.973	0.107	**0.017**	0.586
Rest	0.591	0.993	0.289	0.267	0.496	0.657	0.502	0.128	0.446	0.859	0.686	0.224	**0.002**	**0.002**
Drink	0.750	0.384	**0.051**	0.745	0.510	**0.010**	0.980	0.303	0.899	**0.040**	0.425	0.133	0.225	0.707
Drugs	0.751	0.942	0.679	0.720	0.500	0.247	0.587	0.482	0.581	0.696	0.508	0.383	0.200	0.682
Cigarette	0.857	0.621	0.933	0.984	0.719	0.781	0.140	0.551	0.944	0.155	0.552	0.297	0.408	0.711
Anxiety	0.449	0.689	0.480	**0.024**	**0.045**	0.098	0.082	0.218	0.080	0.086	0.416	0.454	0.796	0.866
Depression	0.866	0.251	0.431	0.044	0.128	0.451	0.592	0.288	0.091	**0.007**	0.588	0.224	0.654	0.422

*^1^MMSE, Mini Mental State Examination.*

*^2^FDT, Five Digits Test.*

*^3^Flex, Flexibility.*

^4^FCR, Rey Complex Figures. *The values in bold indicate a significant p-value in the analyses.

People with CD4 200 > showed worse performance in the attentional processes of choice (χ^2^ = 8.63; *p* = 0.035) and inhibition (χ^2^ = 10.87; *p* = 0.012). People with lower education showed more deficits in cognitive screening (KW = 18.08; *p* = 0.006), lower result in matrix reasoning (KW = 15.11; *p* = 0.019) and in the attentional processes of counting (KW = 18.61; *p* = 0.005), alternating (KW = 15.18; *p* = 0.019) and inhibition (KW = 13.87; *p* = 0.037). People with lower income showed worse performance in the attentional process of reading (KW = 5.81; *p* = 0.055) and higher frequency of HAND (KW = 7.54; *p* = 0.023).

Females performed worse in problem solving (χ^2^ = 10.49; *p* = 0.033) and visual perception and planning (χ^2^ = 16.40; *p* = 0.003). People self-referred as brown-skinned performed worse in the attentional processes of reading (KW = 9.34; *p* = 0.053) and counting (KW = 9.95; *p* = 0.041). People with worse perceptions about diet showed worse visual perception and planning (KW = 10.67; *p* = 0.030). People who did not practice physical activity showed worse attentional performance for reading (KW = 8.45; *p* = 0.038).

In multivariate analyses (Table 4) higher income was shown to reduce the chance of HAND (OR = 0.33), explaining the variation in the data by 11%. Waking up well-rested on more days (OR = 0.63) and perceiving better quality in their own diet (OR = 0.62) reduced the chance of pathological anxiety. These factors explained 20% of the variance in the anxiety data.

**TABLE 4:** Multivariate analyses for HAND, pathological anxiety, and screening for depression.

**HAND**						
	**OR**	**CI 95%**	***p***	**Wald**	***R* ^2^**	**Hosmer–Lemeshow**
Income	0.33	0.14 – 0.77	0.011	6.46	0.11	0.589
**PATHOLOGICAL ANXIETY**						
Wake up well-rested	0.63	0.47–0.83	0.001	10.75	0.20	0.411
Better diet quality	0.62	0.39–0.98	0.042	4.15		
**DEPRESSION**						
Gender female	7.73	3.02–19.77	<0.001	18.23	0.40	0.811
Education	0.74	0.56–0.98	0.036	4.41		
Wake up well-rested	0.61	0.44–0.84	0.003	8.84		

Being female increased the chance of depression results by 7-fold (OR = 7.73), while higher education (OR = 0.74) and waking up well-rested on more days (OR = 0.61) reduced the chance of the disorder. These three variables explained 40% of the variance in the depression data.

## Discussion

This study identified cognitive deficits and emotional symptoms in most of the HIV+ elders investigated. Screening for HAND was present in 89.3% of the sample. The high presence of isolated cognitive deficits or HAND in HIV+ people has been identified independent of age and education (DeVaughn et al., 2015; Wallace et al., 2017) and related to loss of functional independence, poorer quality of life, and lower adherence to ART (Luther and Wilkin, 2007; Heaton et al., 2011; DeVaughn et al., 2015; Fialho et al., 2016).

The worst cognitive scores were perceived in information processing speed, visual memory, attentional alternation, and visual perception. Deficits related to memory, attention, processing speed, and problem-solving ability are the most frequently observed in HIV+ people (Kalil et al., 2009; Cattie et al., 2012; DeVaughn et al., 2015; Nascimento et al., 2015; Antwerpes et al., 2020). Távora et al. (2016) indicate that such deficits are usually identified later in HIV+ elderly, as they take longer to get the diagnosis and start follow-up, which can worsen the impact of these problems.

Depression is the most common psychiatric complication associated with HIV (Caliari et al., 2017; Fialho et al., 2017) and was widely observed in the sample. A possible explanation for the frequency of this comorbidity is the difficulties with the stigma about the diagnosis, problems in affective-sexual relationships, and social exclusion, aspects that mainly affect HIV+ women (Reis et al., 2017). In the HIV+ elderly population, it may be especially important to differentiate depressive disorders from cognitive disorders, as similar symptoms can lead to underdiagnosis of depression and neglect of treatment (Nascimento et al., 2015).

The high anxiety perceived in the sample corroborates findings of previous studies associating anxiety and depression (Nogueira and Seidl, 2016). In the population with HIV, it is observed that the infection tends to increase the anxiety symptoms of people who were already anxious (Nogueira and Seidl, 2016). But other factors also contribute to the elevation of anxiety in these people, including concerns about the progression of the disease, fear of infecting other people and fear of social exclusion (Nogueira and Seidl, 2016).

When assessing the factors associated with HAND, only the income was shown as an independent factor, which went against the study hypotheses, but may be a marker of the profile of the HIV+ population in Brazil. The sociodemographic profile observed in this study shows similarity with the profile of HIV+ Brazilians of other age groups, being marked by people with low education, low income, and a predominance of men (Pinheiro, 2016; Oliveira et al., 2017; Brasil, 2020). A large part of the elderly in the country is in a situation of social vulnerability, which leads them to search less for health professionals, have more underreported pathologies, thus, when they are evaluated, their conditions tend to be more severe or advanced (Nascimento et al., 2015; Cerqueira and Rodrigues, 2016), which may help understand the relationship between income and HAND observed in this study.

The perception about better quality of diet and waking up well-rested on more days reduced the chance of pathological anxiety in HIV+ elderly. The relationship between better diet and emotional state has been previously identified in other populations (Firth et al., 2020), but is an aspect that needs to be better investigated in people with HIV. Similarly, the relationship between sleep, the perception of being well-rested, and lower levels of anxiety and depression has also been previously identified and related to both functioning of the dorsal anterior cingulate cortex of the brain (Klumpp et al., 2017) and with adoption of adaptive coping strategies to deal with concerns (Nogueira and Seidl, 2016).

Factors associated with depression were being female, education, and waking up well-rested. The higher risk for depression among women has been previously identified in several populations (Nanni et al., 2015; Fialho et al., 2017), and there are biological and social role accumulation-related explanations to explain its influence. Another previously perceived association is that of depression with low education, showing the burden of worse living conditions on mental health (Nascimento et al., 2015; Athar et al., 2017).

Our study also has limitations. First, because it was cross-sectional, we were not able to infer causality for the associations found. Another limitation was the absence of a control group, which meant that the results were only compared with the normalized values of the tests and with deficit estimates in the literature for elderly Brazilians. The absence of a control group also prevents us from knowing the specific impact of HIV on the observed deficits, as we do not know what the performance of elderly people with the same socioeconomic conditions without HIV would be when responding to the tests. In addition, our sample size was relatively small and comes from a single service and our analysis will need to be replicated in larger cohorts to confirm the generalizability of our findings. Future research, with larger sample, control group, and that investigate others clinical aspects may help to identify the factors associated with cognitive deficits and emotional symptoms of the HIV+ elderly.

The Brazilian HIV+ ages 50 and older increase in the last two decades, thus, more research is needed to identify neuropsychological deficits and emotional symptoms in this population. Our study suggests that elderly HIV+ are at risk for cognitive deficits, pathological anxiety, and depression. The importance of income, education, quality of food and waking up rested on cognitive and emotional aspects, and indicating the greatest risk of women for depression, showing the need to include these aspects in the evaluation and treatment of this population. Future research will be able to identify whether HIV+ elderly people are at greater risk of cognitive deficits or emotional problems when compared to elderly people without this pathology, investigate the influence of other life and health conditions on cognitive abilities and help to understand the role of perceiving oneself rested in emotional symptoms in HIV+ older people.

## Data Availability Statement

The raw data supporting the conclusions of this article will be made available by the authors, without undue reservations, upon justified request.

## Ethics Statement

The studies involving human participants were reviewed and approved by Institutional Review Board of Federal University of Triângulo Mineiro. The patients/participants provided their written informed consent to participate in this study.

## Author Contributions

SB contributed to the study concept and design, data analysis, and study supervision, and wrote the manuscript. KS contributed to the study concept and design, and data collection. Both authors contributed to the article and approved the submitted version.

## Conflict of Interest

The authors declare that the research was conducted in the absence of any commercial or financial relationships that could be construed as a potential conflict of interest.

## Publisher’s Note

All claims expressed in this article are solely those of the authors and do not necessarily represent those of their affiliated organizations, or those of the publisher, the editors and the reviewers. Any product that may be evaluated in this article, or claim that may be made by its manufacturer, is not guaranteed or endorsed by the publisher.
